# Comparative Study of Hirudins and Encoding Genes in *Hirudo nipponia* and *Hirudo tianjinensis*

**DOI:** 10.3390/biology14091250

**Published:** 2025-09-11

**Authors:** Jingjing Yin, Zichao Liu, Yunfei Yu, Anping Wang, Zuhao Huang, Lizhou Tang, Fang Zhao, Gonghua Lin

**Affiliations:** 1 School of Life Sciences, Key Laboratory of Jiangxi Province for Biological Invasion and Biosecurity, Jinggangshan University, Ji’an 343009, China; m15656142610@163.com (J.Y.); yuyunf2023@163.com (Y.Y.); jskwangap@126.com (A.W.); hzhow@163.com (Z.H.); 2 School of Agronomy and Life Sciences, Kunming University, Kunming 650214, China; abclzc@aliyun.com; 3 College of Life Sciences, Jiangxi Normal University, Nanchang 330022, China; tanglizhou@163.com

**Keywords:** *Hirudo nipponia*, *Hirudo tianjinensis*, hirudin, antithrombin activity, genetic variation, gene expression

## Abstract

This study systematically compared the genetic characteristics and functional activities of hirudins and their encoding genes between *Hirudo nipponia* (Hnip1–3) and *Hirudo tianjinensis* (Htia1–3) through bioinformatics analysis, recombinant protein eukaryotic expression, and activity assays. The results revealed 42 nucleotide variation sites and 27 amino acid variation sites across both species. All six genes were expressed and significant pairwise differences between genes were detected within each species. All hirudins were identified as secretory proteins, with Hnip2, Hnip3, and Htia1 showing higher docking scores with thrombin. Four recombinant proteins (Hnip1, Hnip2, Htia1, and Htia2) exhibited antithrombin activity, with Hnip1 displaying the strongest activity. No significant differences were observed in the sequence variation, gene expression, physicochemical properties, predicted three-dimensional structures, or antithrombin activity of hirudins between the two leech species. This may stem from substantial heterogeneity in the genetic makeup and functional characteristics of distinct hirudins within each species, ultimately reducing the statistical power of these interspecific comparisons. Integrating gene expression profiles with recombinant protein activity assessments revealed that *H. nipponia* hirudins exhibit superior antithrombotic potency compared to those of *H. tianjinensis*. This study establishes a theoretical foundation for medicinal leech resource development and provides critical data for innovative antithrombotic drug discovery.

## Simple Summary

Hirudins are key functional molecules enabling leeches to counteract host coagulation responses, forming the material basis for their medicinal applications. This study compared the genetic characteristics and functional activities of hirudins and their encoding genes between two medicinal leeches *Hirudo nipponia* (Hnip1–3) and *Hirudo tianjinensis* (Htia1–3) through high-throughput sequencing, bioinformatics analysis, recombinant protein eukaryotic expression, and activity assays. Results revealed 42 nucleotide variation sites and 27 amino acid variation sites across both species, comprising 24 genotypes and 23 protein types. All six genes were expressed, with *Hnip2* and *Htia2* showing higher expression levels. All six recombinant hirudins were successfully obtained; four of them (Hnip1, Hnip2, Htia1, and Htia2) exhibited antithrombin activity, with Hnip1 displaying the strongest activity. The two leech species showed no significant differences in hirudin traits—including sequence variation, gene expression, physicochemical properties, predicted three-dimensional structures, or antithrombin activity. This likely results from substantial heterogeneity in genetic makeup and functional characteristics among distinct hirudins within each species, undermining the statistical power of interspecific comparisons. A comprehensive analysis revealed *H. nipponia* hirudins exhibit superior overall antithrombin potency compared to *H. tianjinensis*.

## 1. Introduction

Cardiovascular diseases refer to ischemic or hemorrhagic disorders, occurring in the heart or brain, which are caused by hyperlipidemia, blood viscosity, and atherosclerosis. Globally, approximately 20 million people die annually from various cardiovascular diseases, making it the leading cause of mortality [1,2]. Thrombosis constitutes a critical factor in the occurrence and progression of cardiovascular diseases [3]. Leeches are commonly used animal-derived medicinal materials for preventing and treating thrombotic diseases, containing dozens of antithrombotic proteins such as hirudin, decorsin, antistasin, and destabilase [4]. It is worth noting that most antithrombotic proteins are highly variable between species. For example, the number, sequence, and function of hirudins are highly differentiated among closely related species [5,6] or even within a single species [7]. These proteins serve as key functional molecules enabling leeches to counteract host coagulation responses, forming the material basis for their medicinal applications, thus holding significant research and development values. Hirudin, a small molecular protein comprising approximately 65 amino acids, represents the most effective thrombin-specific inhibitor discovered to date [8,9]. Thrombin is a key enzyme in the final stages of blood clot formation; it cleaves fibrinogen to form insoluble fibrin strands, which polymerize into the structural mesh of a clot, and also activates platelets and other coagulation factors [10]. Hirudin forms an exceptionally stable non-covalent complex through binding with thrombin [11,12], thereby directly blocking this critical step in coagulation, and stands as the representative pharmacological active component in leeches. Hirudins are encoded by multigene families in the leech genome [13]. The number of hirudin genes varies across species, and through evolution, these genes have undergone duplication, recombination, and mutation, generating substantial genetic variation. The anticoagulant activities of different genes can differ by thousands of times [14].

Medicinal leeches in the genus *Hirudo* have been utilized for therapeutic procedures for thousands of years. Due to cultural and historical differences, there are obvious differences in the use of *Hirudo* species between eastern and western countries [15]. In Europe, medicinal leeches have been widely employed since the 17th century to treat various inflammatory conditions and enhance surgical success rates, with *Hirudo medicinalis* Linnaeus, 1758 being the most commonly used species. In China, *Hirudo nipponia* Whitman, 1886 is extensively used in traditional Chinese medicine (orally administered in clinical practice) to treat diverse diseases, particularly thrombosis-related disorders such as stroke and coronary heart disease [16]. Due to morphological and behavioral similarities among *Hirudo* species and lagging taxonomic research, certain species have been misidentified and used interchangeably. For example, molecular studies have shown that European medicinal leeches, although usually marketed as *H. medicinalis*, comprise a complex of at least three species: *H. medicinalis* sensu stricto, *Hirudo verbana* Carena, 1820, and *Hirudo orientalis* Utevsky & Trontelj, 2005 [17]. Interestingly, RT-PCR screening of salivary gland tissue revealed that one or two of the three known hirudin variants (HV1-3) identified from *H. medicinalis* could not be detected in *H. verbana* and *H. orientalis* [5], though it remains unclear whether this difference affects their therapeutic efficacy in blood-feeding therapies.

*H. nipponia* is a renowned medicinal blood-sucking leech [18], commonly found in natural water bodies across East Asia, including China, Japan, Mongolia, and the Russian Far East. Its hosts encompass numerous amphibians, reptiles, mammals, and humans [19]. *H. nipponia* is the only blood-sucking leech species recorded in the Pharmacopoeia of the People’s Republic of China, where it is described as a therapeutic agent for blood stasis, amenorrhea, edema, cerebral hemorrhage, hemiplegia, and trauma [20]. Recognized as one of the most relevant medicinal leech species in traditional Chinese medicine texts [21], it has been utilized for medical and therapeutic purposes for over two millennia [16]. *Hirudo tianjinensis* Wang, 2022, a close relative of *H. nipponia*, exhibits distinct differences in color patterns, reproductive systems, and *COI* sequences [22]. Historically, *H. tianjinensis* has been misidentified and used interchangeably with *H. nipponia*. According to our investigations, the majority of *H. nipponia* specimens in domestic medicinal markets are actually *H. tianjinensis*. Our recent comparative genomics studies of *H. nipponia* and *H. tianjinensis* revealed considerable genetic variations in hirudin genes even between these closely related species [23]. Unfortunately, however, no study has been conducted to date comparing the pharmacological efficacy and antithrombotic effects between the two leech species.

After the first report of the complete amino acid sequence of hirudin from the European medicinal leech (*H. medicinalis*) [24], researchers have subsequently identified other subtypes or variants of hirudin in many medicinal leech species. For example, three hirudin variants (HV1, HV2, and HV3) were reported in *H. medicinalis*, and six hirudins (HM1, HM2, HM3, HM4, P6, and P18) were identified in *Hirudinaria manillensis* Lesson, 1842 [5]. Research has also expanded to species like *H. verbana* [5] and *Hirudo troctina* Johnson, 1816 [25]. Hirudins have also been found in *Hirudo nipponia* Whitman, 1886 and the non-blood-sucking *Whitmania pigra* Whitman, 1884, some of which have been confirmed as potent thrombin inhibitors [7,26]. It is noteworthy that some proteins, despite sharing high homology with hirudin, have been experimentally shown to lack anticoagulant activity and are termed hirudin-like factors (HLFs) [5,27]. Due to their potent anticoagulant activity, both natural hirudin, its recombinant products, and its analogues (hirulogs) have been clinically applied to prevent and treat various thrombotic diseases, particularly venous thrombosis and disseminated intravascular coagulation, as well as to prevent arterial thrombosis after surgical procedures and blood coagulation during hemodialysis [28].

Although hirudin has been extensively studied, the mechanisms underlying the evolutionary diversity of its gene/protein family remain unresolved. All currently known species within the genus *Hirudo* exhibit hematophagous properties [29]; undeniably, hirudin’s anticoagulant function is essential for their survival. Intriguingly, significant interspecies variations exist in hirudin’s gene copy number, expression patterns, sequence divergence, and functional activity among *Hirudo* species. Despite most hirudins sharing three conserved domains (a short N-terminal sequence blocking thrombin’s active site, a central globular domain stabilized by three disulfide bonds, and an elongated C-terminal tail inhibiting thrombin’s fibrinogen-binding site, not considering signal peptide), the relationship between sequence variation and antithrombin activity remains poorly characterized [25,27]. Consequently, this high variability necessitates comparative interspecies studies, particularly among phylogenetically proximate species, to elucidate hirudin’s genetic architecture and functional dynamics. Such research is indispensable for deciphering the molecule’s evolutionary trajectory and unlocking its therapeutic potential.

This study systematically compares the genetic characteristics of hirudin and its encoding genes in *H. nipponia* and *H. tianjinensis* by integrating high-throughput sequencing and bioinformatics analysis. Furthermore, through recombinant protein eukaryotic expression and in vitro functional activity validation, the research comprehensively evaluates and compares the functional activities of hirudin proteins from both leech species, thereby assessing their relative superiority and inferiority in anticoagulant efficacy. The research will provide a crucial theoretical foundation for exploring active components in medicinal leeches, while offering key scientific insights for the modernization of traditional Chinese medicine and the development of innovative pharmaceuticals.

## 2. Materials and Methods

### 2.1. Materials

The live specimens of *H. nipponia* and *H. tianjinensis* were collected in July 2023 from Baodi County, Tianjin, China, with the GPS coordinate of (E 39.47°, N 117.48°). In order to minimize the influence of environmental factors, we chose to collect both leeches in the same location (within 5 km). For each species, twelve individuals were randomly selected to ensure statistical robustness for population-level genomic analyses, balancing biological variability detection with logistical constraints typical of non-model organism studies. Anterior tissues including the salivary gland, hirudin is primarily secreted, were dissected. Total DNA was extracted from each leech using the DNeasy Blood and Tissue Kit (QIAGEN). Concurrently, total RNA was isolated from the head tissues using the TRIzol RNA extraction kit (Thermo Fisher Scientific Inc., Waltham, MA, USA) and purified with the RNeasy Mini Kit (Qiagen, San Diego, CA, USA). DNA quality was verified via spectrophotometry (Nanodrop: A260/A280 ≥ 1.8; A260/A230 ≥ 2.0) and electrophoresis (1% agarose gel), ensuring high molecular weight and absence of degradation. RNA integrity was confirmed using an Agilent Bioanalyzer (RIN ≥ 7.0 for all samples), and purity was assessed (Nanodrop: A260/A280 ≥ 2.0; A260/A230 ≥ 1.8). Qualified DNA and RNA extracts were used to construct libraries (around 350 bp) using Illumina-specific reagents. DNA libraries (350 bp insert size) were prepared using the NEBNext Ultra II FS DNA Kit, while stranded RNA-Seq libraries were generated with the NEBNext Ultra II RNA Kit. Insert size distribution was validated via Bioanalyzer (Agilent 2100, Santa Clara, CA, USA), and library quantification used Qubit dsDNA HS Assay. Whole-genome resequencing and transcriptome (RNA-Seq) sequencing were performed on the BGISeq platform (paired-end 150 bp). Raw sequencing data were processed with fastp v0.20.0 [30] to remove adapters and low-quality regions, generating clean reads for subsequent bioinformatics analysis.

### 2.2. Sequence Extraction

The clean reads from genome resequencing were de novo assembled using Megahit v1.2.9 [31] to generate contigs sequences for each sample. Similarly, the clean reads from transcriptome sequencing were de novo assembled using Trinity v2.9.0 [32] to obtain unigenes sequence files for each sample. BUSCO (Benchmarking Universal Single-Copy Orthologs) v.4.1.4 [33] with the eukaryota_odb10 database was used to assess the completeness of the genome and transcriptome assembly. Using six published *hirudin* genes [23] as bait sequences (nominated as *Hnip1*–*3* and *Htia1*–*3* for *H. nipponia* and *H. tianjinensis*, respectively) (Supplementary File S1), homologous sequences were retrieved from the unigenes files through BLAST v2.13.0+ [34]. The coding region sequences were then extracted after alignment using MEGA v11.0.13 [35]. For genes or samples with low expression levels where complete coding regions could not be obtained from unigenes files, each exon sequence along with its flanking ~50 bp regions was used as bait to retrieve homologous sequences from genome contigs via BLAST. The exon regions were subsequently extracted following alignment with MEGA software.

### 2.3. Variant Site Statistics

The coding region sequences of each gene were merged into individual FASTA files. The aligned sequences were translated using MEGA for subsequent comparative analysis. DnaSP v6 [36] was employed to calculate the number of variable sites (VS) and haplotype numbers (HN) for each gene. Watterson’s Theta diversity (WD) was computed using DAMBE v7.3.5 [37]. Gene sequences were translated into amino acid sequences using MEGA, followed by statistical analysis of amino acid sequence variation sites, haplotype numbers, and Watterson’s Theta diversity index using DAMBE.

### 2.4. Gene Expression Analysis

Using all coding region sequences obtained from whole-genome structural annotation as a reference template, sequence indexes were constructed using Salmon v1.0.0 software [38]. Transcriptomic reads from each sample were then aligned to the indexed files, with a core parameter setting of k-mer value = 31. The transcripts per million (TPM) values of each hirudin gene’s coding region sequences were calculated to represent their relative expression levels in individual samples. Expression differences between species and among different hirudin genes within the same species were analyzed using SPSS v25. One-Sample Kolmogorov–Smirnov test revealed that the expression levels of most hirudin genes significantly deviated from normal distribution across individuals (*p* < 0.05), indicating the appropriateness of non-parametric statistical approaches. The overall expression levels of hirudin genes in *H. tianjinensis* and *H. nipponia* were compared using the Mann–Whitney U test. Expression differences between each gene from *H. nipponia* and that from *H. tianjinensis* were similarly analyzed with Mann–Whitney U tests. To assess expression variation among *hirudins* within species, we first performed the Several-Related-Samples test (Friedman test) to detect significant differences in overall expression levels across hirudin genes. Where significant differences were identified (*p* < 0.05), pairwise comparisons between individual hirudin genes were subsequently conducted using Two-Related-Samples test (Wilcoxon signed-rank tests).

### 2.5. Protein Biochemical Property Analysis

The six *hirudin* gene sequences were translated into protein sequences (nominated as Hnip1–3 and Htia1–3 for *H. nipponia* and *H. tianjinensis*, respectively) using MEGA. The signal peptide regions were determined comprehensively using the SignalP v6.0 online tool (https://services.healthtech.dtu.dk/services/SignalP-6.0/, accessed on 20 April 2025) combined with amino acid sequence alignment information. Subcellular localization of the proteins was performed using CELLO v.2.5 (http://cello.life.nctu.edu.tw/, accessed on 22 April 2025). After removing the signal peptide regions with MEGA software (Supplementary File S2), the isoelectric point, instability index, and grand average of hydropathicity (GRAVY) for each protein were predicted using the ProtParam online tool (https://web.expasy.org/protparam/, accessed on 24 April 2025).

### 2.6. Protein Three-Dimensional Structure Analysis

Using the three-dimensional structure file of the archetypal *H. medicinalis* hirudin HV1 (PDB accession number: 1HRT) as a reference, three-dimensional structure homology prediction was performed for six hirudin proteins from two leech species using the PyMod v3.0 plugin [39] in the PyMOL v2.5.4 software package [40]. The FATCAT v2.0 [41] was employed to compare the three-dimensional structures of hirudin from *H. nipponia* and *H. tianjinensis* with the archetypal *H. medicinalis* hirudin. Molecular docking was performed using the ZDOCK server with bovine thrombin (PDB: 1HRT) and human thrombin (PDB: 1DOJ) as receptors, and six hirudin proteins from two leech species as ligands. Default parameters were applied (grid spacing: 1.2 Å; angular step size: 6°), with interface residues identified post-docking using a 10 Å cutoff. Based on HV1-thrombin binding site data [42], we selected five key thrombin residues (SWGEG, conserved in both bovine and human thrombin) and five N-terminal residues from each hirudin variant for docking.

### 2.7. Pichia Pastoris Eukaryotic Expression

Six cDNA (with signal peptide region removed) plasmids were ordered from Sangon Biotech (Shanghai, China). The plasmids were amplified by culturing *E. coli* in LB liquid medium with overnight shaking incubation on a shaker. Plasmid DNA was extracted using a plasmid DNA miniprep kit from Sangon Biotech (Shanghai, China) by collecting and lysing the bacterial cells. The plasmid DNA was linearized using SpeedyCut *Sac* I restriction enzyme and further purified with a purification kit from Sangon Biotech (Shanghai, China). The purified linearized plasmid DNA was chemically transformed into *Pichia pastoris* GS115 competent cells and plated on YPDZ agar plates containing 0.25% Geneticin (a suitable antibiotic for pPIC9K vectors we used). The plates were incubated at 30 °C for 3–5 days until yeast colonies emerged. Subsequent antibiotic selection was performed sequentially on YPDZ plates containing 0.5%, 1%, and 2% Geneticin. The yeast cells were inoculated into BMGY medium for scale-up culture and then transferred to BMMY medium for methanol-induced fermentation. Finally, salt precipitation and desalting procedures were carried out to isolate recombinant proteins encoded by the six target genes. The desalted exogenous expressed protein solution was placed in a freeze dryer for lyophilization. Then, it was dissolved in approximately 5 mL of phosphate-buffer solution. Finally, the concentration of the protein solution was detected and recorded using a Nano spectrophotometer.

### 2.8. Antithrombin Activity Assay

The antithrombin activity of protein aqueous solutions in each group was determined according to the thrombin titration method (as specified in the leech monograph requirements of the 2020 edition of the Pharmacopoeia of the People’s Republic of China [20]). We used the protein aqueous solution expressed by *Pichia pastoris* without insertion of any target gene as the control. Qualitative analysis: 100 μL of the test protein aqueous solution and 200 μL of fibrinogen (10 mg/mL) were added to a 2 mL centrifuge tube. They were mixed thoroughly and incubated in a 37 °C water bath for 1 min. Then 50 μL of thrombin solution (50 U/mL, NIH-units) was added and incubated in the 37 °C water bath for another 10 min. The results were observed and recorded. Complete coagulation indicated no antithrombin activity in the test protein solution, while incomplete coagulation confirmed the presence of antithrombin activity. Quantitative analysis: A volume of the test protein aqueous solution (less than 100 μL) was added to a 2 mL centrifuge tube and adjusted the total volume to 100 μL using pH 7.4 phosphate buffer. 200 μL of fibrinogen (10 mg/mL) was added. They were mixed thoroughly and incubated in a 37 °C water bath for 1 min. Then 50 μL of thrombin solution (50 U/mL) was added and incubated in the 37 °C water bath for 10 min. The results were observed and recorded. If no coagulation occurred, the volume of the test protein solution (supplemented to 100 μL with buffer) was gradually reduced until a semi-coagulated state (30–70% solidified) was observed.

The minimum volume of the test protein solution required to achieve this state was recorded and the corresponding antithrombin activity for each protein sample was calculated with the following formula: Antithrombin Activity (ATU/mg) = (50 × 50)/(V × C) × K. Here, the first 50 refers to the concentration of the thrombin solution (50 U/mL); the second 50 refers to the volume of the thrombin solution (50 μL = 0.05 mL); V represents the volume consumed (μL) to achieve “half-coagulation” in the test protein aqueous solution; C represents the concentration of the test protein aqueous solution (mg/mL); and K represents the correction coefficient (generally taken as 1 according to the requirements of the Pharmacopoeia). To ensure sensitivity and reliability, we employed a stepwise approach: initial screening used 20–50 μL increments of test protein aqueous solution to detect anticoagulant activity and estimate its approximate dose range; subsequent refinement with 10 μL steps narrowed the dosage range; finally, 2 μL increments determined the precise reaction volume. The “half-coagulation” state was deemed effective when maintained >3 min without reversion upon mixing reversal.

We tested each protein sample in triplicate. The differences in antithrombin activity between different species and between different hirudin proteins within the same species were analyzed using SPSS v25 software. A One-Sample Kolmogorov–Smirnov Test revealed that the antithrombin activity of all hirudin proteins significantly deviated from a normal distribution among individuals (*p* < 0.01). Therefore, non-parametric methods were appropriate for statistical analysis of this data. A Two-Independent-Samples Test (Mann–Whitney U test) was used to examine whether there were significant differences in antithrombin activity of hirudin proteins between different species. A Several-Related-Samples Test (Friedman test) was used to determine if there were significant differences in antithrombin activity overall between different hirudin proteins within the same species. If significant differences were found, a Two-Related-Samples Test (Wilcoxon signed-rank test) was then used to conduct pairwise comparisons of antithrombin activity between the different hirudin proteins within the same species.

## 3. Results

### 3.1. Intraspecific Variation in the Hirudin Genes and Their Proteins

Through second-generation sequencing, we obtained 12 genome resequencing datasets and 12 transcriptome sequencing datasets for each species, respectively. The average clean data size per sample exceeded 20 Gb for genomes and 10 Gb for transcriptomes. The Q20 values for the vast majority of both genome and transcriptome datasets were greater than 97%. The average (mean ± SD) contig N50 of the assembled genomes and unigene N50 of the transcriptomes were 3954.8 ± 1030.5 bp and 3261.0 ± 231.6 bp, respectively. BUSCO analysis revealed that 87.2 ± 17.0% and 98.3 ± 0.7% of BUSCOs were captured for the genome and transcriptome assemblies, respectively. The statistics of the raw data and assemblies are presented in Supplementary Table S1. Mann–Whitney U test indicated no significant difference in the proportion of BUSCOs captured between the genome (*Z* = −1.850, *p* = 0.064) and transcriptome (*Z* = −0.058, *p* = 0.954) assemblies of *H. nipponia* and *H. tianjinensis*, ensuring comparability between the two species.

All six *hirudin* genes of *H. nipponia* and *H. tianjinensis* were identified across 12 samples (Supplementary Files S3 and S4). The coding sequence lengths of these genes ranged between 190–270 bp, with no pseudogene detected. After aligning, a total of 42 variable sites at the DNA level and 27 variable sites at the protein sequence level were identified, respectively. Specifically, *H. nipponia* exhibited 19 and 15 variable sites, respectively, at DNA and protein levels, while *H. tianjinensis* displayed, respectively, 23 and 12 variable sites. For *H. nipponia*, *Hnip1* displayed the highest number of variable sites, followed by *Hnip3*, and with *Hnip2* showing the least. In *H. tianjinensis*, *Htia3* contained the most variable sites, followed by *Htia2*, while *Htia1* exhibited no variation (Table 1).

**Figure 1:**
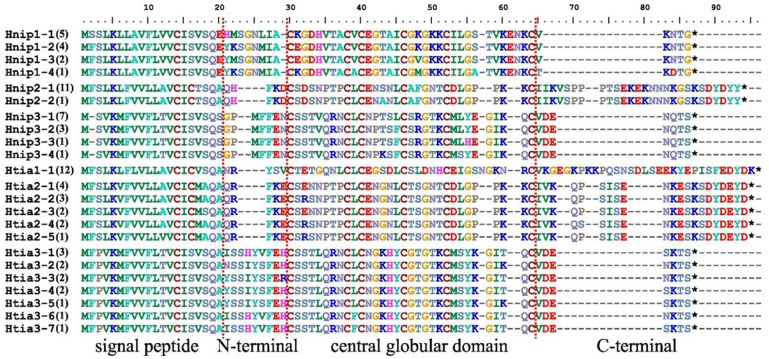
The aligned haplotypes of hirudin protein in *Hirudo nipponia* and *Hirudo tianjinensis*. The four sequence regions (signal peptide, N-terminal, central globular domain, and C-terminal) were separated by red line of dashes; asterisks denote stop codons; the frequency of each haplotype is indicated in parentheses.

A total of 24 DNA sequence haplotypes and 23 protein sequence haplotypes were detected, with the majority of variations between different haplotypes occurring in functional regions. Specifically, *H. nipponia* and *H. tianjinensis* exhibited 10 and 14 DNA sequence haplotypes, and 10 and 13 protein haplotypes (Figure 1), respectively. This trend was similarly reflected in the Watterson’s Theta diversity indices at both DNA and protein levels (Table 1). Mann–Whitney U test showed that, no significant differences (*Z* ≤ −0.218, *p* ≥ 0.507) were observed in any of the variables listed in Table 1 between the two leech species. This may stem from the heterogeneity in these characteristics of distinct hirudins within each species, ultimately reducing the statistical power of these interspecific comparisons.

### 3.2. Gene Expression

**Figure 2:**
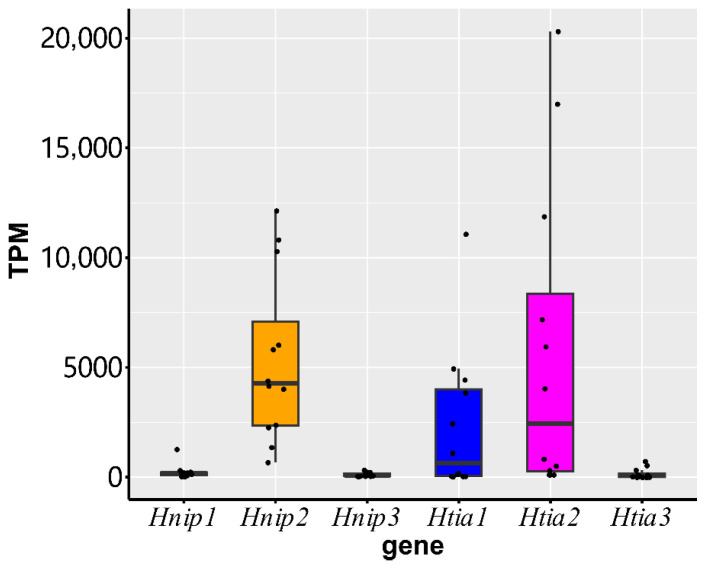
Relative expression levels of hirudin genes based on transcriptomic analysis (TPM: transcripts per million). Boxes represent interquartile ranges (IQR), horizontal lines indicate median values, and whiskers extend to the minimum and maximum values within the non-outlier range.

Analysis of relative expression levels based on transcriptomic data showed that the mean ± SD TPM values for *Hnip1*, *Hnip2*, *Hnip3*, *Htia1*, *Htia2*, and *Htia3* genes were 250.4 ± 333.3, 5365.5 ± 3829.7, 125.9 ± 96.3, 2362.4 ± 3331.1, 5700.7 ± 7119.9, and 166.9 ± 240.2, respectively (Figure 2 and Supplementary Table S2). The overall mean ± SD TPM values in *H. nipponia* and *H. tianjinensis* were 5741.7 ± 3282.8 and 8230.0 ± 4976.1, respectively, indicating that the overall expression level of *hirudin* genes in *H. tianjinensis* was slightly higher than that in *H. nipponia*. However, the Mann–Whitney U test revealed no statistically significant difference (*Z* = −0.462, *p* = 0.644) between them.

The Mann–Whitney U test indicated significant differences for the following interspecies pairs: Hnip1 vs. Htia2 (*Z* = −2.540, *p* = 0.011), Hnip2 vs. Htia1 (*Z* = −2.194, *p* = 0.028), Hnip2 vs. Htia3 (*Z* = −4.099, *p* < 0.001), and Hnip3 vs. Htia2 (*Z* = −3.233, *p* = 0.001). No significant differences were observed in the remaining five pairs (*p* > 0.05). Friedman tests revealed significant differences in expression levels among the three *hirudins* in both *H. nipponia* (*χ*^2^ = 19.5, *p* < 0.001) and *H. tianjinensis* (*χ*^2^ = 24.0, *p* < 0.001). Subsequent Wilcoxon signed-rank tests showed significant differences between all pairwise comparisons of hirudins within *H. nipponia* (*Z* ≤ −1.961, *p* ≤ 0.05) and within *H. tianjinensis* (*Z* = −3.059, *p* = 0.002), indicating substantial intraspecies expression heterogeneity.

### 3.3. Protein Biochemical Property

The results of the biochemical property analysis of the proteins are shown in Table 2. Subcellular localization analysis showed that all six hirudins were localized extracellularly. The Mann–Whitney U test revealed no significant differences in the isoelectric point (pI), instability index, and water solubility between the two leech species (*Z* = −1.091, *p* = 0.275). Notably, these parameters varied significantly among different proteins within each species. For example, each of the two leech species had one alkaline protein (pI > 7) and two acidic proteins (pI < 7). Furthermore, the instability index and hydrophilicity index of the different hirudins between the two leech species exhibited a more than one-fold difference between their maximum and minimum values.

### 3.4. Protein Three-Dimensional Structure

Based on the three-dimensional structural comparison using the FATCAT v2.0 software, all six hirudins showed significant structural similarity (*p* < 0.001) to the archetypal *H. medicinalis* hirudin (PDB No: 1HRT) (Table 3). Among them, Hnip1, Hnip3, and Htia3 exhibited higher similarity than the other three hirudins. Mann–Whitney comparisons revealed no significant differences in the statistical metrics (*p* value and *RMSD*) between the two leech species (*Z* = −0.655, *p* = 0.513). However, within each species, certain metrics still showed substantial variation among different proteins. Molecular docking results using ZDOCK demonstrated inconsistent binding scores between these hirudins and bovine/human thrombin. For bovine thrombin, Hnip3 and Htia2 achieved the highest docking scores, while Hnip2, Hnip3, and Htia1 showed relatively higher scores for human thrombin (Table 3). Mann–Whitney analysis indicated no significant differences in docking scores between the two species (bovine thrombin: *Z* = −0.655, *p* = 0.513; human thrombin: *Z* = −1.091, *p* = 0.275). However, within the same species, docking scores varied markedly among different proteins, with the maximum value to be at least twice the minimum value.

### 3.5. Antithrombin Activity

**Figure 3:**
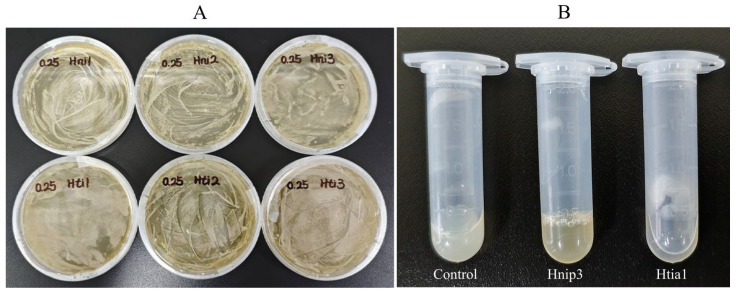
The experimental images. (**A**) Yeast clones of the six hirudins; (**B**) Experimental examples of antithrombin activity assay (Control, clotted; Hnip3, clotted; Htia1, not clotted).

We synthesized six recombinant hirudin proteins using the eukaryotic expression technology of *Pichia pastoris*. The purified linearized plasmid DNA was successfully chemically transformed into *Pichia pastoris*, as evidenced by the appearance of yeast colonies on YPDZ agar plates containing 0.25% Geneticin (final concentration) (Figure 3A). Rapid extraction of yeast genomic DNA followed by PCR amplification and agarose gel electrophoresis confirmed the presence of corresponding bands (Supplementary Figure S1). Ultimately, we successfully obtained exogenously expressed proteins for all six target genes. The raw concentrations of the expressed proteins Hnip1, Hnip2, Hnip3, Htia1, Htia2, and Htia3 were determined to be 1.347, 3.191, 1.954, 9.235, 15.207, and 17.152 mg/mL, respectively.

The antithrombin activity of each protein was detected using the thrombin titration method, expressed as the number of thrombin activity units inhibited per unit mass of protein sample under specific experimental conditions, typically denoted as ATU/mg. Based on Figure 3B, when the 2 mL centrifuge tube was placed horizontally on the table, the control sample (protein expressed by *Pichia pastoris* without any target gene insertion) completely clotted. Similarly, Hnip3 (which lacks antithrombin activity) also fully clotted, while Htia1 (possessing antithrombin activity) remained liquid. This demonstrates a clear distinction in experimental observations between the presence and absence of antithrombin activity. Table 4 summarizes the antithrombin activity measurements, including means and standard deviations, for hirudin proteins from *H. nipponia* and *H. tianjinensis* across three replicate assays. The results revealed significant antithrombin activity in Hnip1, Hnip2, Htia1, and Htia2, while no activity was detected in Hnip3 or Htia3. For *H. nipponia*, Hnip1 displayed the highest activity at 220.754 ATU/mg, more than three times higher than that of the second-ranked Hnip2. In *H. tianjinensis*, Htia1 exhibited the strongest activity, surpassing the second-ranked Htia2 by over twofold. The consistently low standard deviation values relative to their means across replicate measurements demonstrate high assay sensitivity and reliability.

Within *H. nipponia*, Friedman test analysis showed that the antithrombin activity among its three hirudin proteins differed significantly overall (*Chi-Square* = 6.000, *df* = 2, *p* < 0.05). Furthermore, Wilcoxon signed-rank tests found that the antithrombin activity differed significantly between each pairwise comparison of the hirudin proteins (*Z* < −1.96, *p* < 0.05). Similarly, within *H. tianjinensis*, Friedman test analysis showed that the antithrombin activity among its three hirudin proteins also differed significantly overall (*Chi-Square* = 6.000, *df* = 2, *p* < 0.05). Wilcoxon signed-rank tests also revealed significant differences in antithrombin activity between each pairwise comparison of the hirudin proteins (*Z* < −1.96, *p* < 0.05). However, Mann–Whitney comparison revealed no significant difference in the antithrombin activity of hirudin proteins between different species (*Z* = −1.619, *p* = 0.105). Again, this may stem from heterogeneity among distinct hirudins within each species, which reduces the statistical power of interspecific comparisons.

## 4. Discussion

Leeches belong to the phylum Annelida, class Clitellata, and subclass Hirudinea, in which many species feed on mammalian blood. To ensure successful feeding within tens of minutes, leeches secrete a series of bioactive substances such as anticoagulants, thrombolytics, and anti-inflammatory agents to counteract the host’s physiological hemostatic processes [43], endowing them with significant medical and pharmaceutical value. The *Pharmacopoeia of the People’s Republic of China*, one of the most crucial components of China’s pharmaceutical regulations, stipulates that materials not listed in it are theoretically prohibited from medicinal use. The current edition of the Pharmacopoeia recognizes three leech species—*H. nipponia*, *W. pigra*, and *Whitmania acranulata* Whitman, 1886—as the legal sources of “Shuizhi” for medicinal purposes. As *H. tianjinensis* has been confirmed as a distinct species [44], regulations require it to be treated as an adulterant of *H. nipponia* and banned from medicinal use. However, due to historical reasons, the pharmaceutical market has long failed to distinguish between *H. nipponia* and *H. tianjinensis*. Hirudin, the most representative active component in leeches, warrants comparative studies on both the protein and its encoding genes in sympatrically distributed *H. nipponia* and *H. tianjinensis*, as such research holds significant value for the development of related pharmaceuticals.

The hirudins Hnip1 (Hnip_HV5), Hnip2 (Hnip_V3a), Htia1 (Hnip_V1a) and Htia2 (Hnip_V2) were already described and (three of them) functionally characterized before [7]. Based on our data and its comparison with the prior characterization, here is a breakdown of the key similarities, differences, and implications. Our study robustly confirms the core findings of the prior study regarding which hirudins from *H. nipponia* (Hnip1 and Hnip2) and *H. tianjinensis* (Htia1 and Htia2) possess antithrombin activity, and which do not (Hnip3 and Htia3). The quantitative differences observed (especially Hnip1’s high activity and Htia2’s relatively lower activity compared to Htia1) compared to potentially less pronounced differences in the prior study could be due to differences in protein production (e.g., different expression system) or assay methodology/sensitivity. Additionally, our research findings reveal that no significant differences were observed in the sequence variation, gene expression, physicochemical properties, predicted three-dimensional structures, or antithrombin activity of hirudins between the two leech species. This may stem from substantial heterogeneity in the genetic makeup and functional characteristics of distinct hirudins within each species, ultimately reducing the statistical power of these interspecific comparisons. Interestingly, these variations appear to follow no discernible pattern. For instance, while the conventional view suggests that functionally critical genes should exhibit higher conservation, this seems only partially applicable. In *H. tianjinensis*, the antithrombin activity of Htia1, Htia2, and Htia3 progressively decreases while their conservation levels correspondingly increase. Conversely, in *H. nipponia*, Hnip1 demonstrates the highest activity but shows the weakest conservation. Our previous phylogenetic analyses indicate that the six genes do not form species-specific clades. Instead, *Hnip2* clusters with *Htia2*, and *Hnip3* with *Htia3*, forming distinct monophyletic groups. This suggests that the gene duplication and differentiation of the three *hirudin* genes in both species had already occurred in their common ancestor. We therefore propose that the genetic and functional features of different *hirudin* genes within each species have evolved independently. Recent comparative genomic studies have revealed exceptionally high genomic variability in annelids, potentially linked to their elevated recombination rates [45]. Consequently, we hypothesize that the substantial genetic and functional differences among the three hirudin genes/proteins within each species (*H. nipponia* and *H. tianjinensis*) may primarily result from stochastic mutations occurring at distinct chromosomal loci.

Similarly, the activity of hirudin appears to have no direct correlation with its physicochemical properties or three-dimensional structure. Taking *H. nipponia* as an example, Hnip1 exhibits the strongest antithrombin activity, yet its sequence conservation, expression level, and thrombin-binding affinity are not the highest. The observed inconsistency between its antithrombin activity and thrombin affinity is particularly puzzling. Notably, previous three-dimensional structural studies on the interaction between hirudin and thrombin revealed that hirudin interacts with thrombin over an extended area, both within and far from the active site, e.g., of the 65 residues of hirudin of *H. medicinalis*, 27 actually contact thrombin [42]. We hypothesize that variations in the number of thrombin contact sites among different hirudins may result in molecular docking simulations—which typically consider only optimal docking configurations—failing to accurately reflect their overall activity. In other words, compared to other hirudins, Hnip1 may engage with thrombin through more interaction sites, though further evidence is required to substantiate this hypothesis. Molecular docking simulations often struggle to accurately capture the precise energetics and conformational consequences of these specific terminal interactions, particularly the N-terminal insertion. Additionally, post-translational modifications present in the expression system (such as glycosylation in yeast), the structural flexibility of proteins, or the inherent limitations of in silico computational models may also be contributing factors to the inconsistencies between molecular docking predictions and the actual functional activity.

Hirudin achieves its anticoagulant function by forming a non-covalent complex with thrombin through binding [12,46]. Therefore, the expression level of the *hirudin* gene directly affects the synthesis of hirudin protein, thereby influencing its overall activity. Transcriptomic analysis demonstrated non-significant interspecies differences in overall hirudin gene expression, yet revealed significant pairwise differences among hirudin genes within each species, indicating substantial intraspecies expression heterogeneity. In *H. nipponia*, the TPM value of *Hnip2* is significantly higher than those of the other two *hirudin* genes, approximately 20- and 40-fold higher than *Hnip1* (which also has anticoagulant activity) and *Hnip3* (which lacks anticoagulant activity), respectively. Considering that the per-unit activity of the protein products of *Hnip1* and *Hnip2* differs by approximately 3.3-fold, it can be inferred that the *Hnip2* gene plays a decisive role in the antithrombotic function of *H. nipponia*, with *Hnip1* contributing secondarily and *Hnip3* making no contribution. In *H. tianjinensis*, the TPM value of *Htia2* is consistently higher than those of the other two *hirudin* genes, approximately 2- and 30-fold higher than *Htia1* (which also has anticoagulant activity) and *Htia3* (which lacks anticoagulant activity), respectively. Given the approximately 2-fold difference in per-unit activity between the protein products of *Htia1* and *Htia2*, it can be concluded that the *Htia2* gene plays a key role in the antithrombotic function of *H. tianjinensis*, with *Htia1* contributing secondarily and *Htia3* having no effect.

As previously mentioned, the current edition of the *Pharmacopoeia of the People’s Republic of China* designates *H. nipponia* as the official source for the “Shuizhi” medicinal material. The question arises: can *H. tianjinensis* qualify as an alternative origin for “Shuizhi”? Generally speaking, the efficacy of leech-based medicinal materials depends on the unit catalytic activity and the number of anticoagulant proteins. The unit catalytic activity of the proteins can be experimentally determined and calculated, but since we did not perform purification, it is difficult for us to obtain the number of monomeric proteins. Although gene expression levels are not exactly equivalent to protein expression levels, higher transcriptional activity is likely to produce more protein products. Assuming a linear correlation between hirudin gene expression and protein synthesis in both leech species, *Hnip1* expression is approximately 10-fold of *Htia1*, while the per unit activity of Hnip1 is about one-tenth that of Htia1. Thus, the net activities of these two proteins largely counterbalance each other. Given that neither Hnip3 nor Htia3 exhibits detectable activity, the total anticoagulant effect of hirudins in these leeches depends exclusively on Hnip2 and Htia2. Considering *Hnip2* expression is 9-fold greater than *Htia2* with comparable activity per unit protein, we conclude that *H. nipponia* demonstrates superior hirudin-derived anticoagulant activity relative to *H. tianjinensis*.

However, this does not conclusively indicate inferior overall antithrombotic efficacy in *H. tianjinensis*. Beyond hirudin, both leech species harbor multiple bioactive components with anticoagulant, antiplatelet, anti-inflammatory, and thrombolytic properties. Notably, *H. tianjinensis* exhibits a higher gene count for decorsin, eglin, HMEI, and apyrase, which may establish compensatory mechanisms for coagulation inhibition [23]. For our next step, we plan to expand our research to other gene families. We intend to employ a murine arterial/venous thrombosis model to test crude extracts from both species. We will measure thrombus formation and systemic coagulopathy markers, aiming to conduct a more systematic comparative evaluation of the antithrombotic activity between the two leech species.

It should also be noted that in our previous research on *H. manillensis*, we observed significant variations in genetic diversity and gene expression of hirudins across geographical populations, which are likely influenced by regional environmental factors [47]. To rule out such environmental effects, we selected *H. nipponia* and *H. tianjinensis* samples with sympatric distribution for this study. However, due to environmental pollution and overfishing, such sympatric populations have currently only been identified in a single location. Consequently, we collected only 12 samples for each species, a number lower than the generally recommended minimum sample size (e.g., 20 for genetic diversity studies on amphibians [48]). Nevertheless, we believe the findings of this study remain reasonably reliable. First, probably due to the overall highly variable genomes of annelids as mentioned above, a considerable number (*N* = 42) of intraspecific nucleotide variant sites were identified across the six hirudin genes. However, the extent of genetic variation differed considerably among the hirudin genes; for example, *Htia3* possessed the most variant sites, whereas *Htia1* showed none. Additionally, while the expression levels of each *hirudin* genes exhibited high standard deviations among individuals, significant pairwise differences among these genes within each species were observed. These distinctly different properties among the genes provided reliable information for comparative analysis. Furthermore, the raw genomic and transcriptomic data demonstrated high quality and completeness: Q20 values exceeded 97% for both datasets, and the transcriptome BUSCO score reached 98%. Moreover, all assembled hirudin sequences contained complete coding regions without any instances of frameshift mutations or premature stop codons, supporting the authenticity of the observed hirudin sequence variations. Of course, it remains important to acknowledge that the limited sample size and narrow geographical scope in this study may lead to an underestimation of genetic variation, potentially affecting the reliability of the conclusions. Future studies will expand sampling across a broader geographic range to gain a better understanding of the differences in hirudins between the two leech species.

## 5. Conclusions

This study systematically compares the in silico characteristics and in vitro functional activity of hirudin in *H. nipponia* and *H. tianjinensis*. No significant differences were observed in the sequence variation, gene expression, physicochemical properties, predicted three-dimensional structures, or antithrombin activity of hirudins between the two leech species. This may stem from substantial heterogeneity in the genetic makeup and functional characteristics of distinct hirudins within each species, ultimately reducing the statistical power of these interspecific comparisons. Weighted sum analysis of gene expression and recombinant protein activity revealed that *H. nipponia* hirudins exhibited significantly superior comprehensive antithrombin potency compared to *H. tianjinensis*. The research will provide a crucial theoretical foundation for exploring active components in medicinal leeches, while offering key scientific insights for the modernization of traditional Chinese medicine and the development of innovative pharmaceuticals.

## Figures and Tables

**Table 1:** Intraspecific variation in hirudin genes and proteins.

Hirudin CDS	Length of Sequence	Coding Sequence			Protein Sequence		
		VS	HN	WD	VS	HN	WD
*Hnip1*	204	13	4	0.03476	10	4	0.08021
*Hnip2*	252	2	2	0.00794	1	2	0.01190
*Hnip3*	198	4	4	0.01054	4	4	0.03162
*Htia1*	270	0	1	0.00000	0	1	0.00000
*Htia2*	234	8	6	0.01390	5	5	0.02857
*Htia3*	207	15	7	0.02958	7	7	0.04141
Total	1365	42	24	—	27	23	—

Note: VS, number of variable sites; HN, number of haplotypes; WD, Watterson’s Theta diversity.

**Table 2:** Biochemical properties of hirudin proteins of *Hirudo nipponia* and *Hirudo tianjinensis*.

Factor	PSL (Reliability)	pI	II	GRAVY
Hnip1	Extracellular (3.686)	8.87	26.28	−0.036
Hnip2	Extracellular (4.243)	6.72	35.27	−1.070
Hnip3	Extracellular (3.165)	6.21	16.54	−0.437
Htia1	Extracellular (4.250)	4.84	39.29	−1.113
Htia2	Extracellular (4.142)	5.22	65.38	−1.135
Htia3	Extracellular (3.390)	7.79	20.78	−0.390

Note: PSL, Protein Subcellular Localization; pI, isoelectric point; II, Instability index; GRAVY, grand average of hydropathicity.

**Table 3:** Comparison of the three-dimensional structures and molecular docking of hirudin proteins from *H. nipponia* and *H. tianjinensis*.

Protein	Structural Comparison		Docking Score	
	*p*-Value	*RMSD*	Bovine Thrombin	Human Thrombin
Hnip1	1.44 × 10^−12^	0.22	899.209	933.955
Hnip2	1.25 × 10^−5^	3.37	922.550	2089.823
Hnip3	1.56 × 10^−12^	0.36	2136.436	1754.866
Htia1	5.83 × 10^−10^	1.47	1609.944	1728.753
Htia2	3.80 × 10^−8^	2.07	1661.418	824.945
Htia3	2.00 × 10^−12^	0.39	1241.655	1059.792

**Table 4:** Antithrombin activity of hirudin proteins of *H. nipponia* and *H. tianjinensis*.

Hirudin	Antithrombin Activity (ATU/mg)			
	Repeat 1	Repeat 2	Repeat 3	Mean ± SD
Hnip1	212.766	222.222	227.273	220.754 ±7.364 ^a^
Hnip2	66.225	66.667	67.568	66.820 ± 0.684 ^b^
Hnip3	0.000	0.000	0.000	0.000 ± 0.000 ^c^
Htia1	16.892	17.123	16.667	16.894 ± 0.228 ^A^
Htia2	7.267	7.184	7.353	7.268 ± 0.085 ^B^
Htia3	0.000	0.000	0.000	0.000 ± 0.000 ^C^

Note: different superscript letters indicate significant within-group differences (Wilcoxon Signed-Rank test, *p* < 0.05); the lower and upper case letters represent different meanings, where the lower case letters represent *H. nipponia*, and the upper case letters represent *H. tianjinensis*.

## Data Availability

Data are contained within the article and Supplementary Files.

## Supplementary Materials

The following supporting information can be downloaded at: https://www.mdpi.com/article/10.3390/biology14091250/s1, File S1: Hirudin CDSs in reference genomes of two leech species; File S2: Hirudin protein sequences for the experiment; File S3: Hirudin CDSs obtained in 12 *H. nipponia* samples; File S4: Hirudin CDSs obtained in 12 H. tianjinensis samples; Table S1: Statistics for raw data and assemblies from 12 *H. nipponia* and 12 *H. tianjinensis* samples; Table S2: TPM values of hirudin genes of 12 *H. nipponia* and 12 *H. tianjinensis* samples; Figure S1: Agarose gel electrophoresis of PCR-amplified hirudin genes.

## Abbreviations

CDS	coding sequence
VS	number of variable sites
HN	number of haplotypes
WD	Watterson’s Theta diversity
TPM	transcripts per million
PSL	protein subcellular localization
pI	isoelectric point
II	instability index
GRAVY	grand average of hydropathicity
