# Supplementary material for: Comparative Study of Hirudins and Encoding Genes in Hirudo nipponia and Hirudo tianjinensis
Source: Biology (Basel). 2025 Sep 11;14(9):1250. doi: 10.3390/biology14091250 (PMC12466982; doi:10.3390/biology14091250)
Supplement: Supplementary file 1 [file biology-14-01250-s001.zip › Supplementary tables and figures.pdf]

Table S1. Statistics for raw data and assemblies from 12 *Hirudo nipponica* (Hnip01–12) and 12 *Hirudo tianjinensis* (Htia01–12) samples.

Sample	Genome reads		Genome contigs			Transcriptome reads		Transcriptome unigenes		
	Total bases (bp)	Q20 (%)	Length (bp)	N50 (bp)	BUSCO (%)	Total bases (bp)	Q20 (%)	Length (bp)	N50 (bp)	BUSCO (%)
Hnip01	26,186,163,900	97.54	246,051,015	3856	91.6	15,194,187,300	98.37	174,790,457	3346	98.5
Hnip02	29,605,714,500	97.59	203,693,305	3515	84.7	13,660,886,400	98.43	165,082,226	3287	98.1
Hnip03	29,497,441,800	97.45	209,960,320	3481	88.5	16,430,313,900	98.52	190,633,425	3418	98.5
Hnip04	35,185,041,300	97.42	268,295,849	4420	89.8	11,948,125,200	98.18	167,232,402	3250	98.1
Hnip05	31,791,888,900	97.62	911,627,147	4581	95.3	17,492,232,300	98.27	198,979,945	3494	99.3
Hnip06	31,243,429,200	97.35	205,185,479	3849	87.1	16,762,178,700	98.17	189,356,364	3564	99.6
Hnip07	33,481,794,300	97.59	308,889,884	3099	90.2	16,598,533,200	98.11	193,813,466	3466	97.3
Hnip08	87,841,491,000	99.42	283,694,267	4388	89.4	18,563,650,500	98.36	192,194,542	3361	97.7
Hnip09	30,417,014,100	97.70	245,972,845	3077	91.7	15,911,626,200	98.33	184,616,120	2901	98.1
Hnip10	27,506,355,600	97.69	258,283,135	3837	91.0	16,822,623,300	98.25	147,748,221	2887	98.0
Hnip11	28,231,474,200	97.43	255,175,732	3780	88.7	17,676,426,600	98.48	183,307,438	2971	98.1
Hnip12	38,011,598,700	97.25	291,904,499	2197	92.1	13,469,020,800	98.33	174,191,699	2937	98.8
Htia01	29,439,477,600	97.47	246,116,831	4434	91.8	11,353,329,600	98.04	212,967,577	3415	97.7
Htia02	27,165,483,900	97.27	240,134,214	5371	82.1	13,737,315,000	98.29	215,278,297	3619	99.3
Htia03	57,633,712,200	98.98	726,952,138	508	8.7	12,672,407,400	98.25	179,291,324	3246	97.2
Htia04	31,015,462,800	97.42	242,194,730	4418	92.6	12,700,632,600	98.25	185,881,211	3324	98.5
Htia05	28,526,565,300	96.95	241,226,413	4198	93.0	14,891,660,700	98.27	215,804,246	3461	99.2
Htia06	25,530,747,000	96.98	234,350,221	4701	92.6	10,888,592,100	98.51	191,058,459	3302	98.8
Htia07	24,834,194,100	97.05	238,200,200	4483	92.2	17,808,436,200	98.26	229,661,934	3583	97.3
Htia08	20,452,065,600	96.97	218,734,815	5247	93.3	16,220,365,500	98.37	186,915,151	3293	98.1
Htia09	23,597,848,800	96.94	247,092,621	4140	92.1	16,633,669,800	98.38	231,665,908	3140	98.5
Htia10	21,555,123,900	96.87	241,841,545	4378	92.6	16,046,313,300	98.45	221,111,316	3124	99.2
Htia11	22,891,289,100	97.02	251,414,473	3809	91.8	15,435,792,600	98.33	195,120,274	2930	98.8
Htia12	26,612,806,200	99.44	249,852,227	5148	91.0	14,989,803,600	98.40	174,634,662	2946	97.3

Table S2. TPM values of hirudin genes of 12 *H. nipponia* and *12 H. tianjinensis* samples.

H. nipponia	*Hnip1*	*Hnip2*	*Hnip3*	*H. tianjinensis*	*Htia1*	*Htia2*	*Htia3*
Hnip01	313.42	10821.22	255.73	Htia01	47.70	120.27	1.60
Hnip02	89.95	2382.68	43.76	Htia02	131.65	832.87	32.68
Hnip03	170.69	4020.72	233.12	Htia03	4947.49	20314.06	729.96
Hnip04	145.08	5821.82	59.15	Htia04	2449.45	5953.16	114.68
Hnip05	187.60	4159.55	48.45	Htia05	54.79	312.78	3.53
Hnip06	243.35	2270.00	54.70	Htia06	45.66	117.48	3.34
Hnip07	36.21	6031.26	123.63	Htia07	1103.56	4041.86	106.50
Hnip08	1275.59	12145.48	332.14	Htia08	11079.95	17006.66	99.36
Hnip09	95.91	10294.82	53.63	Htia09	23.88	121.11	11.46
Hnip10	36.38	678.52	73.49	Htia10	177.83	518.71	31.65
Hnip11	202.71	1365.66	133.98	Htia11	4440.28	11880.92	327.82
Hnip12	207.38	4393.88	99.19	Htia12	3846.87	7188.55	540.43

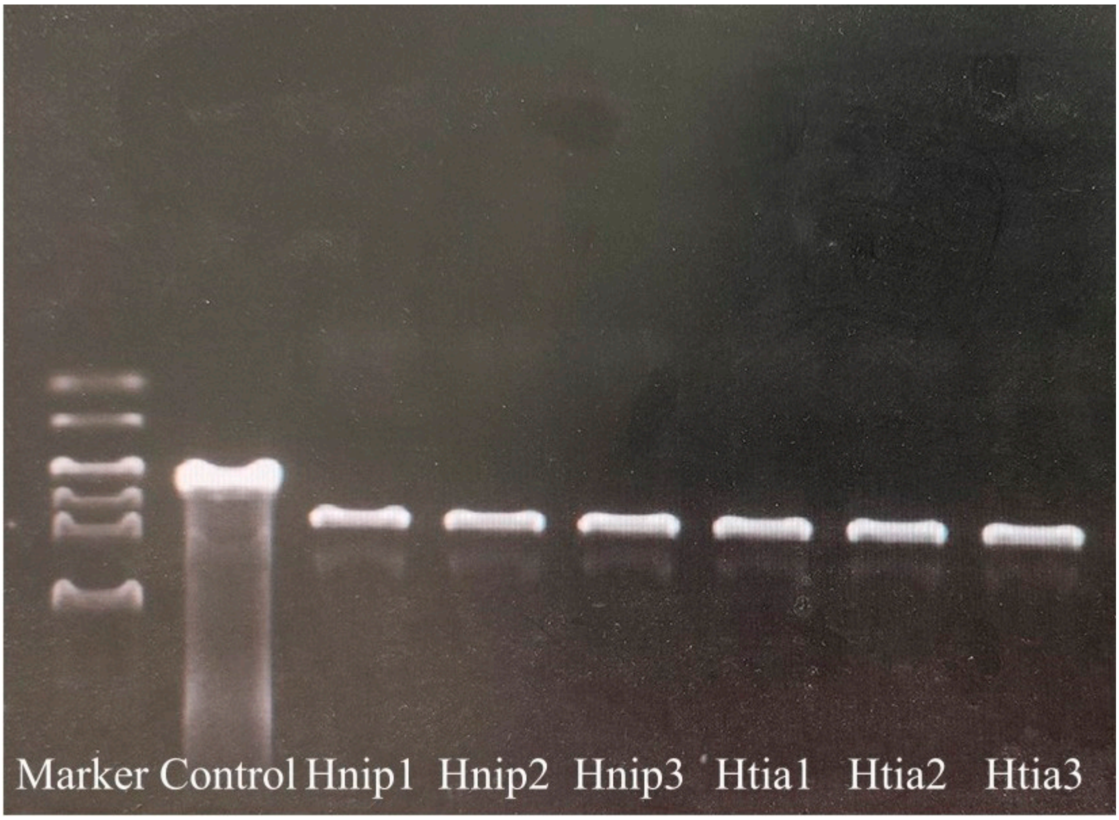

Figure S1. Agarose gel electrophoresis of PCR-amplified hirudin genes.
